# Healthy Dwelling: Design of Biophilic Interior Environments Fostering Self-Care Practices for People Living with Migraines, Chronic Pain, and Depression

**DOI:** 10.3390/ijerph19042248

**Published:** 2022-02-16

**Authors:** Dorothy Day Huntsman, Grzegorz Bulaj

**Affiliations:** 1 Dayhouse Studio, Salt Lake City, UT 84106, USA; dorothy@dayhousestudio.com; 2 OMNI Self-Care, LLC, Salt Lake City, UT 84111, USA; 3 College of Pharmacy, Department of Medicinal Chemistry, University of Utah, 30 South 2000 East, Salt Lake City, UT 84112, USA

**Keywords:** built environment, restorative, biophilia, salutogenesis, home care, non-pharmacological interventions, self-management, mental health, neurological disorders

## Abstract

The benefits of biophilic interior design have been recognized by healthcare facilities, but residential environments receive relatively less attention with respect to improving the health of people living with chronic diseases. Recent “stay-at-home” restrictions due to the COVID-19 pandemic further emphasized the importance of creating interior spaces that directly and indirectly support physical and mental health. In this viewpoint article, we discuss opportunities for combining biophilic interventions with interior design, fostering disease-specific self-care. We provide examples of designing residential spaces integrating biophilic interventions, light therapy, relaxation opportunities, mindfulness meditation, listening to music, physical activities, aromatherapy, and quality sleep. These modalities can provide the clinical benefits of reducing migraine headaches and chronic pain, as well as improving depressive symptoms. The disease-specific interior environment can be incorporated into residential homes, workplaces, assisted-living residences, hospitals and hospital at home programs. This work aims to promote a cross-disciplinary dialogue towards combining biophilic design and advances in lifestyle medicine to create therapeutic interior environments and to improve healthcare outcomes.

## 1. Introduction

People living with chronic diseases experience both debilitating disease symptoms and diminished health-related quality of life. Chronic pain, migraines and depression are some of the leading causes of disability worldwide [1]. Currently available therapies for chronic diseases include pharmaceutical drugs and non-pharmacological interventions such as behavioral therapies, physical therapies and digital therapeutics. However, despite progress in developing new medical treatments, the global burden of neurological diseases has increased [2]. 

A sedentary lifestyle significantly contributes to the causes and symptoms of chronic disease [3]. On average, people in the US, Canada and Germany spend 15–17 h per day indoors [4]. “Stay-at-home” restrictions, due to the COVID-19 pandemic, further decreased physical activity, having an adverse impact on mental health [5]. Lifestyle modifications and self-care practices can help patients to improve their health [6,7,8]. For the purpose of this work, self-care is defined as “the ability of individuals, families and communities to promote health, prevent disease, maintain health and to cope with illness with or without the support of a healthcare provider” (definition by the World Health Organization). Self-care practices include quality sleep, relaxation, mindfulness practices, listening to music, physical activity, healthy nutrition, aromatherapy, stress management and connecting with nature. 

Residential environments are often overlooked as a complementary means to improve therapeutic outcomes [9]. The emerging field of biophilic design has been focusing on therapeutic indoor environments for decades [10,11,12,13,14,15,16,17]. The biophilic design philosophy is derived from an inherent need for humans to connect with nature in order to stay in physical and mental balance [12,18]. The concept of biophilia suggests that the tendency of humans to affiliate with nature has, in part, a genetic basis, hence the inherent need for connection [10,18]. The benefits of nature connectedness include relaxation, stress relief, lower blood pressure and heart rate, decrease in chronic pain, improvement in cognitive functioning, increased positive emotions, and reduced fatigue, aggression and sadness [19,20,21]. 

Biophilic design principles are generally organized into three categories: nature in the space, nature of the space and natural analogues [12,22]. Nature in the space is creating a visual connection with nature; for instance, using vegetation (plants, flowers and trees) both outside and inside a residential space. Nature of the space is the imitation of natural processes, such as the circadian rhythms through lighting patterns, and spatial compositions found in nature, such as creating a reading nook to give a sense of refuge and safety. Natural analogues are representations of the textures, geometries, materials, colors, shapes and patterns found in the natural world that provide an indirect sense of the great outdoors. The 2015 report by Kellert and Calabrese describes biophilic design principles as providing “repeated and sustained engagement with nature” and focus “ on human adaptations to the natural world that over evolutionary time have advanced people’s health, fitness and well-being” [11]. 

In addition to residential spaces, biophilic design is applicable to healthcare facilities [22] and workplaces [23,24]. For example, biophilic design was recently utilized in long-term residential care for people with dementia [25]. Other studies suggest that it can be optimized for people with post-traumatic stress disorder [26], autism spectrum disorders [27,28] and to improve mental health outcomes [29]. The interior design opportunities are further illustrated by reports of biophilic design to mitigate physical and cognitive decline in aging adults [30], and for those living with disabilities [31]. In this viewpoint article, we describe opportunities to expand biophilic design by integrating indoor spaces fostering diverse self-care practices. We provide a rationale for this cross-disciplinary approach to creating therapeutic interior environments for people living with specific chronic diseases. 

## 2. Integrating Biophilic Design and Self-Care to Create Therapeutic Interior Environments

**Figure 1:**
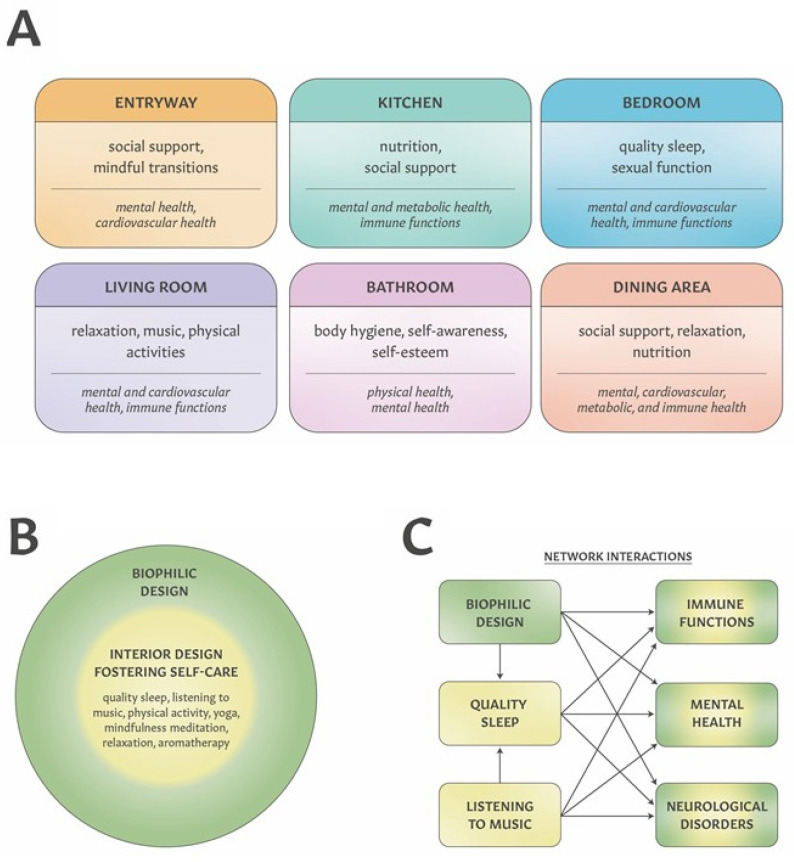
Graphical representation of transforming residential indoor spaces into therapeutic interior environment opportunities by integrating biophilic design and disease-specific self-care. (**A**) Examples of the relationship between residential indoor spaces, daily functions and health. (**B**) The concept of therapeutic interior environments created by integrating biophilic design with spaces fostering self-care practices. (**C**) Potential benefits of integrating biophilic design and self-care are presented as a network of mutually beneficial interconnections improving health-related outcomes. As an example, both biophilic design and listening to music can improve quality sleep, while all three (biophilic elements, quality sleep and music) have positive effects on mental health, neurological functions and the immune system.

Housing is recognized as an important determinant of human health [32], providing multiple opportunities to transform residential indoor spaces into therapeutic environments. Figure 1A illustrates possible relationships between specific residential spaces and health-related domains (e.g., affective states, cardiovascular, metabolic, and immune functions). For example, a living room that promotes relaxation (e.g., through biophilic features, soundscapes to deliver music, furniture to foster relaxation and meditation) and physical activities (exercise or yoga practice) can support mental and cardiovascular health. Since quality sleep impacts mental, cardiovascular, neurological and immune functions [33,34,35,36], the design of a bedroom that supports sleep hygiene, resting and relaxation (e.g., through biophilic elements, using clean lines, calming colors, cultivating a feeling of intimacy, providing dimmable lighting using “zero-blue” bulbs) can further support people living with chronic diseases. 

Transforming residential spaces into therapeutic interior environments can be accomplished through combining biophilic design with design features fostering self-care, as shown in Figure 1B. Biophilic design is applicable to any of the residential indoor spaces, including the living room, bedroom, kitchen, dining room, etc. Judicious design of biophilic features throughout the residence can provide a continuum of exposure to indoor nature. Figure 1C illustrates the prospects of creating mutually beneficial interactions when integrating biophilic design and indoor environments supporting disease-specific self-care. The network of diverse physiological responses to such therapeutic interior environments is supported by accumulating studies showing that nature-based interventions and listening to music can improve depressive symptoms and pain (e.g., [19,37,38,39,40,41,42,43,44] while also improving sleep [45,46,47,48]. It is noteworthy that all three—nature-based interventions, listening to music and quality sleep—can also positively impact immune functions [33,49,50]. In subsequent sections, we discuss how a combination of interior features (including biophilic design, furniture, lighting, aromatherapy and soundscapes) and self-care practices can be combined to create a therapeutic environment for people living with migraines, chronic pain and depression. 

## 3. Therapeutic Interior Design for People with Chronic Diseases

**Figure 2:**
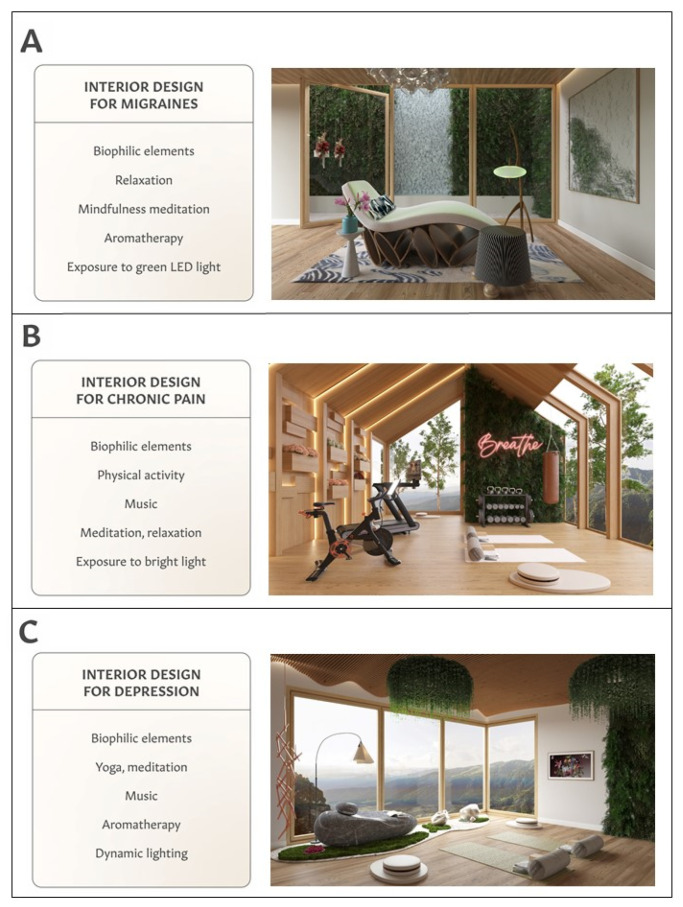
Designing therapeutic interior environments for people with migraines (**A**), chronic pain (**B**) and depression (**C**). Disease-specific interior designs are based on studies summarized in Table 1, Table 2 and Table 3.

As described in the Introduction, research shows that biophilic design can yield positive effects on health and well-being [12,13,17]. Biophilic interventions have been shown to improve relaxation, reduce stress and depressive symptoms, and provide pain relief [43,44,51,52,53,54,55,56,57,58,59,60,61]. Herein, we show examples of residential spaces combining biophilic design principles with beneficial self-care components individualized for persons living with migraines, chronic pain and depression (Figure 2). To provide a rationale for incorporating specific self-care practices into residential indoor spaces, we summarize the clinical benefits of relaxation, mindfulness meditation, listening to music, physical activities, aromatherapy and quality sleep (Table 1, Table 2 and Table 3). In addition, we describe how advances in light therapy can inspire the design of lighting systems to reduce migraine headaches, chronic pain and depressive symptoms. These descriptions are intended to promote cross-disciplinary discussion on innovative interior design for people living with chronic diseases. 

### 3.1. Designing Therapeutic Interior Environments for People with Migraines

Migraines are one of the most prevalent chronic diseases associated with significant disability. Among diverse precipitating factors for migraines, stress is considered the top trigger [62]. Recent reviews on non-pharmacological self-management of migraines suggest that several self-care modalities can reduce pain intensity and headache-related disability [63]. Lifestyle recommendations for people with migraines include stress management and sleep hygiene [64]. Table 1 summarizes the rationale for integrating diverse modalities, which can directly and/or indirectly provide health benefits for people living with migraines. Combining biophilic design with additional interior features fostering relaxation can lead to lower stress levels, thus supporting migraine prophylaxis. Exposure to green LED light and aromatherapy with lavender essential oils may lead to a reduction in headache frequency and severity [65,66]. The mechanisms by which lavender essential oil may improve a migraine include the inhibition of neurogenic inflammation, [66] and promoting relaxation and stress reduction [67,68,69].

**Table 1:** Research evidence on incorporating biophilic interventions and self-care modalities into therapeutic interior environments for people with migraines.

Modality	Studies Supporting Interior Landscapes Integrating Biophilic Interventions and Self-Care
Exposure to nature	Exposure to houseplants and flowers can improve relaxation and reduce stress [51,52,53,54,55,56] Viewing nature indoors promotes relaxation [57] Biophilic interventions can reduce stress [58] Touching white oak wood increases relaxation and calms prefrontal cortex activities [59,60] Exposure to outdoor nature can reduce psychophysiological stress [70,71,72] Nature exposure can reduce both perceived and physiological stress [73]
Green LED light	Exposure to green LED light significantly decreases headache days in migraine patients [65]
Aromatherapy	Aromatherapy with lavender significantly reduces headache severity in migraine patients [74] Inhalation of peppermint essential oil reduces the intensity and frequency of headaches [75]
Relaxation and mindfulness meditation	Relaxation training improves headache frequency and pain severity [76] Mindfulness-based stress reduction decreases migraine days [77] Mindfulness can improve headache-related disabilities and well-being [78] Relaxation and mindfulness meditation are suggested for migraine prophylaxis [79]
Yoga	Yoga practice reduces headache frequency and intensity in migraine patients [80]
Sleep hygiene	Sleep disorders are associated with more frequent and severe migraines [81] Behavioral sleep treatment can reduce headache frequency and intensity [82,83]
Nutrition	Magnesium can be used as prophylactic treatment of migraines [84,85] Ginger can reduce headache severity in migraine patients [86]

As illustrated in Figure 2A, the customized interior design for people with migraines includes biophilic elements and features supporting migraine self-care. Ambient conditions are complex and varied, but familiar and comfortable. Sounds, smells and textures remind one of being outside in nature. All elements are intended to bring about a feeling of relaxation and calm. The biophilic elements consist of dynamic lighting, plants, fresh flowers, biomorphic and fractal shapes, a water-scape, the judicious use of wood and high-quality air filtration. While not shown, an addition of the Frame Smart TV (Samsung^®^) would provide nature-inspired art, music and opportunities for guided meditation. Features supporting migraine self-care include an LED lamp delivering both green light and aromatherapy, as well as a comfortable lounge chair supporting meditation, relaxation and stress reduction. 

Varied and dynamic lighting—a biophilic aspect—is appointed in this case to accommodate a person with migraines’ specific light sensitivities; for example, dimmable lighting, indirect natural lighting opportunities and ambient cove lighting. The combination of these lighting applications adds to the sense of calm, relaxation and tranquility reminiscent of the indirect qualities of a natural environment. Plants and fresh flowers are applied as additional biophilic elements to support relaxation and positive emotions [55,56,87]. 

### 3.2. Designing Therapeutic Interior Environments for People with Chronic Pain 

Chronic pain is a debilitating disorder, leading to disability and a reduced health-related quality of life. Chronic pain conditions include lower back pain, arthritis pain, cancer pain and neuropathic pain, as well as complex syndromes, such as fibromyalgia. Lifestyle (physical activity, nutrition and smoking) is associated with chronic pain [88,89]. Non-pharmacological modalities for pain management and relief may include physical therapy, yoga, mindfulness meditation, listening to music, sleep hygiene and nature therapy (Table 2). The American College of Physicians recommends physical exercises and yoga as a first-line therapy for lower back pain [90]. It is noteworthy that perioperative exposure to music is associated with the reduced intake of analgesic drugs [91,92]. There is also a growing number of studies indicating that exposure to nature and natural light may provide analgesia and reduce the burden of pain [42,93]. 

**Table 2:** Clinical evidence on incorporating nature exposure and self-care modalities into therapeutic interior environments for people with chronic pain.

Modality	Studies Supporting Interior Landscapes Integrating Biophilic Interventions and Self-Care
Exposure to nature	Exposure to flowers can improve fibromyalgia pain and postoperative pain [43,44] Forest bathing can reduce posterior neck pain [42] Forest bathing can reduce chronic pain and depressive symptoms, while also increasing natural killer (NK) cell activity [49]
Lighting	Exposure to natural sunlight can reduce pain and use of analgesic medications [61] Exposure to morning bright light can reduce lower back pain [94,95] Exposure to home-based morning bright light can improve fibromyalgia pain sensitivity [96] Exposure to green LED light can reduce pain in fibromyalgia patients [97]
Music	Music can reduce chronic pain and the use of pain medications [39,91,92] Music significantly reduces postoperative pain [92,98] Music reduces pain in fibromyalgia patients [99]
Physical activity, yoga and breathing exercises	Physical activity and yoga are recommended by the American College of Physicians clinical guidelines as the first-line therapy for lower back pain [90] Breathing exercises can reduce chronic lower back pain and improve quality of life [100]
Mindfulness meditation	Mindfulness meditation can significantly reduce chronic pain and depressive symptoms [101,102]
Sleep hygiene	Poor sleep quality is associated with increased pain intensity [103,104]
Combination of modalities	A combination of nature exposure, physical activity, education and social support can improve pain and fatigue in fibromyalgia patients [105] A combination of music, relaxation and guided imagery reduces pain in fibromyalgia patients [106,107] Exercise and meditation reduces lower back pain intensity [108]

The therapeutic interior design concept for people with chronic pain includes biophilic elements and features supporting chronic pain self-care (Figure 2B). One of the key features is the room’s natural light and focus on natural surroundings; for example, shade trees with a vista to foster a connection with nature. Other biophilic features consist of natural woods, indoor plants, biomorphic shapes and fresh flowers. The architectural design mimics symmetry and the use of repeating patterns as found in basic nature structures. Artificial smart lighting supports natural circadian rhythm cycles.

A room that provides the opportunity for, and encouragement of, exercise fosters the self-management of chronic diseases. Physical activities are supported by the presence of exercise and wellness equipment while facing a natural landscape and vista, thus integrating exercise and exposure to nature. Multiple yoga mats foster stretching and yoga practice with a companion, bridging physical activity and social support. To enrich this therapeutic environment, an integrated sound system serves as a high-quality music delivery system. Direct sunlight is an important part of designing for chronic pain, while providing exposure to nature and a vantage point can lead to faster stress recovery [109]. The design also includes ambient lighting that holds the capacity to emulate the blue light of the morning sun, and the yellow light of the evening sun, as well as narrow-band green LED light, shown to reduce pain in people with fibromyalgia [97].

### 3.3. Designing Therapeutic Interior Environments for People with Depression 

Depression is a chronic mental disorder for which antidepressant and behavioral therapies have limited efficacy [110]. Depression is often comorbid with other chronic diseases, including chronic pain and neurodegenerative disorders. Living with depression is associated with increased disability and suicide risk [111,112]. Lifestyle medicine for depression includes nutrition, physical exercise and recreation, relaxation and meditation, sleep quality and social support [113]. As summarized in Table 3, there are multiple self-care modalities which can clinically benefit people living with depression. Positive effects of exposure to nature on mental health and well-being are well established [114,115]. The mechanisms by which exposure to nature can modulate affective states include stress reduction and the improvement of cognitive functions [116]. Bright light therapy has been effective in treating depression, including bipolar depression [117]. Recent reviews and meta-analysis studies support listening to music as an effective non-pharmacological intervention for depression [37,38]. Based on the beneficial effects of exposure to nature and self-care modalities (Table 3), we suggest designing an interior environment that would support emotional regulation by increasing both arousal and valence with visual and acoustic stimulation. 

**Table 3:** Clinical evidence on incorporating nature exposure and self-care modalities into therapeutic interior environments for people with depression.

Modality	Studies Supporting Interior Landscapes Integrating Biophilic Interventions and Self-Care
Exposure to nature	Exposure to outdoor nature can reduce depression [41,71] Green space view from a window can reduce risk of depression [118] Exposure to green spaces can affect mental health and well-being [119] Exposure to flowers can reduce depressive symptoms in fibromyalgia patients [44] Exposure to natural environments increases positive affect and decreases negative affect [120,121]
Lighting	Bright light therapy is effective for non-seasonal depression and bipolar depression [117,122,123] Bright light therapy is effective for people with seasonal affective disorder [124,125] Blue-wavelength light therapy can be effective in treating depression [126]
Music	Music reduces depressive symptoms [37,38]
Physical activity and yoga	Physical activity can reduce depressive symptoms [127,128,129] Yoga can reduce depressive symptoms [130]
Mindfulness meditation	Mindfulness meditation can improve depressive symptoms [131,132]
Breathing exercises	Breathing exercises reduce depressive symptoms in patients with major depressive disorder who did not respond to antidepressant medications [133] Breathing exercises can reduce depressive symptoms [134,135]
Aromatherapy	Aromatherapy can improve depressive symptoms [136]
Sleep hygiene	People with insomnia have a higher risk for developing depression [137] Altering circadian rhythms can be linked to major depression [138]
Nutrition	Supplementation with St John’s Wort has significant clinical efficacy in reducing depressive symptoms, including people living with major depressive disorder [139,140] Mediterranean diet is associated with a lower risk for depression [141]

Figure 2C summarizes suggested interior design features for people living with depression. These include biophilic elements such as a fractal ceiling, indoor plants, fresh flower art, natural wood, a biomorphic floor lamp, and floor-to-ceiling windows overlooking a natural landscape and providing direct sunlight. Interior features fostering self-care practices include meditation space, yoga mats, an integrated diffuser in a floor lamp for aromatherapy, and the Frame Smart TV (Samsung^®^) as a delivery system of music, guided meditations and nature-inspired art. All biophilic and other design features encourage relaxation and stress reduction, which can mitigate depressive symptoms [142]. Dynamic lighting for this purpose includes natural bright light, provided by large windows, and cove lighting surrounding the space, for ambient lighting effects that can provide bright light year-round. Two yoga mats are placed to encourage yoga practice and stretching exercises with a companion, thus simultaneously providing social support. 

## 4. Expanding Interior Design Elements for Therapeutic Purposes 

As shown in Figure 2, renderings of therapeutic interior environments emphasize diverse biophilic elements delivered via natural light (through windows and a smart lighting system), the presence of indoor plants and fresh flowers, fractals, soundscapes, biomorphic shapes, natural materials and colors, and vista viewpoints. In addition, the design of therapeutic spaces for people with migraines, chronic pain or depression (Figure 2), can include the incorporation of such indoor features as the Frame Smart TV (Samsung^®^) and a smart lighting system, which can provide the benefits of nature therapy, music therapy and light therapy. Using the Frame Smart TV to provide both biophilic art and a high-quality sound system for music and guided meditation offers innovative ways to expand the application of a TV set as a delivery system of therapies for pain or depression [37,38,39,91,92,98]. Currently, such frame TV models are marketed for their abilities to serve as a standard TV or as décor to display art, when in the “off” position.

Another example of expanding the applications of interior features is to use lighting systems for light therapy purposes. Light therapy has been recognized as treatment for depression or fatigue [123,143,144], while recent studies suggest that specific LED light conditions can provide clinical benefits for people living with migraines, fibromyalgia and chronic pain ([145] and references in Table 1 and Table 2). Specific in-home lighting conditions were used for the treatment of fatigue in people with traumatic brain injury [146,147]. Effects of light exposure on sleep quality are well documented [148,149], while there are direct correlations between the quality of sleep and diverse health conditions [34,35,103,150]. Therefore, lighting conditions which promote sleep hygiene may provide additional health benefits for people living with migraines, insomnia, fibromyalgia, chronic pain or depression.

Rapid advancements in the smart LED lighting systems allow a user to program bright light in the morning to reduce pain, depression and fatigue, or provide green LED light therapy for a reduction in migraine headaches. Currently, health-related light bulbs include those with: (1) eliminated, or reduced, blue color wavelength light, which are marketed as “circadian rhythms bulbs”, “no blue”, “low blue”, “zero blue” or “restful bedtime bulb”, (2) green LED light, marketed as “migraine relief LED light” or “green light for migraine sufferers” by Allay Lamp^®^, and (3) natural light, marketed as “natural sunlight”, “natural daylight light” or “full-spectrum sunlight like”. Such diverse LED technologies are commercially available as “stand alone” bulbs, or as an integrated, smart lighting system, thus facilitating the custom design of lighting within biophilic interior design environments for people with specific medical conditions.

## 5. Advancing Therapeutic Interior Design to Improve Healthcare Outcomes

**Figure 3:**
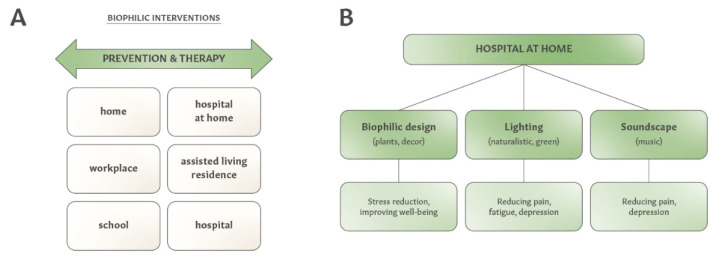
Applications of biophilic interventions and health-related interior landscapes in diverse indoor environments. (**A**) Examples of diverse indoor environments amenable to health-related interior design. (**B**) Examples of therapeutic interior design elements and their clinical benefits for hospital at home care.

With the number of people living with chronic diseases increasing worldwide, there are ongoing needs to innovate and to expand disease prevention and treatments. As illustrated in Figure 3A, creating therapeutic indoor environments is applicable not only to residential homes, but also for hospital rooms, assisted-living facilities and hospital at home (HaH) programs [151]. While biophilic design has been recognized by healthcare facilities [22,152,153,154,155] and assisted-living housing [156], there are no reports of applying health-related interior design for HaH care. The COVID-19 pandemic has renewed an interest in home care services [157,158]; therefore, therapeutic interior design for HaH may offer additional clinical benefits. An example of interior design features for an HaH setting could include biophilic elements, a smart lighting system, and soundscapes delivering relaxing music (Figure 3B). Since the judicious design of lighting can improve quality of sleep, fatigue, depression, migraine headaches and chronic pain, while listening to music can reduce pain and depressive symptoms, these two interior elements alone can provide clinical benefits for people with cancer, COPD and other medical conditions, which are preferred when considering HaH care.

In the U.S. alone, the design of disease-specific therapeutic environments could benefit a significant portion of the 330 million people living in 128 million households. To this end, the Biophilic Design Matrix has been proposed to facilitate the implementation of biophilic attributes by interior designers [22,159,160]. Future prospects for therapeutic interior environments can also include integration with digital health technologies for migraines [161,162], chronic pain [163,164] and depression [165,166]. It is appealing to suggest that integrated healthcare systems such as Sanford Health, Kaiser Permanente, Intermountain Healthcare, Trinity Health and others may consider partnerships with architectural firms and interior design companies to create, validate and implement residential and commercial interior environments for people living with diverse chronic diseases.

We acknowledge the limitations of this viewpoint article, which include diverse levels of evidence to support therapeutic interior design, thus being prone to publication bias. Recent review articles on biophilic design point out research gaps on the relationships between biophilic attributes and health-related outcomes [9,13,16,17,24,160]. To the best of our knowledge, there are only a few reports on combining biophilic interventions with pharmacotherapies [43,61]. With respect to studies on self-care for specific chronic diseases, we include references on systematic review and meta-analysis (SR/MA) when available; however, given the diversity of research evidence for biophilic interventions and individual self-care modalities (ranging from small-group observational and pilot studies, longitudinal studies, narrative reviews, RCTs and SR/MA), presenting such a cross-disciplinary topic as an SR/MA would be difficult at the present time. We emphasize an ongoing need for more research and development projects to evaluate the efficacy and effectiveness of therapeutic interior environments and disease-specific self-care for people living with migraines, chronic pain, depression and other chronic diseases.

## 6. Conclusions

Despite a recognition of the benefits of biophilic design by healthcare facilities, transforming residential indoor spaces into therapeutic environments receives relatively less attention. In this viewpoint article, we discuss the integration of biophilic interventions with indoor features fostering self-care for people living with migraines, chronic pain and depression. We conclude that therapeutic interior design supporting a connection with nature, healthy lifestyle and disease-specific self-care practices offers unique opportunities to improve healthcare outcomes in residential applications and beyond. 

## Data Availability

Not applicable.
